# First study on diversity and antimicrobial-resistant profile of staphylococci in sports animals of Southern Thailand

**DOI:** 10.14202/vetworld.2022.765-774

**Published:** 2022-03-29

**Authors:** Punpichaya Fungwithaya, Kanpapat Boonchuay, Ruethai Narinthorn, Narin Sontigun, Chalutwan Sansamur, Yotsapat Petcharat, Thotsapol Thomrongsuwannakij, Tuempong Wongtawan

**Affiliations:** 1 Akkraratchkumari Veterinary College, Walailak University, Thai Buri, Tha Sala, Nakhon Si Thammarat, Thailand 80160; 2 Centre for One Health, Walailak University, Thai Buri, Tha Sala, Nakhon Si Thammarat, Thailand 80160; 3 Excellence Centre for Melioidosis and Other Microorganisms, Walailak University, Thai Buri, Tha Sala, Nakhon Si Thammarat, Thailand 80160

**Keywords:** antimicrobial resistance, fighting bulls, fighting cocks, riding horses, *Staphylococcus*

## Abstract

**Background and Aim:**

Staphylococci are commensal bacteria and opportunistic pathogens found on the skin and mucosa. Sports animals are more prone to injury and illness, and we believe that antimicrobial agents might be extensively used for the treatment and cause the existence of antimicrobial-resistant (AMR) bacteria. This study aimed to investigate the diversity and AMR profile of staphylococci in sports animals (riding horses, fighting bulls, and fighting cocks) in South Thailand.

**Materials and Methods:**

Nasal (57 fighting bulls and 33 riding horses) and skin swabs (32 fighting cocks) were taken from 122 animals. Staphylococci were cultured in Mannitol Salt Agar and then identified species by biochemical tests using the VITEK^®^ 2 card for Gram-positive organisms in conjunction with the VITEK^®^ 2 COMPACT machine and genotypic identification by polymerase chain reaction (PCR). Antimicrobial susceptibility tests were performed with VITEK^®^ 2 AST-GN80 test kit cards and VITEK^®^ 2 COMPACT machine. Detection of AMR genes *(mecA*, *mecC*, and *blaZ)* and staphylococcal chromosomal *mec* (SCC*mec*) type was evaluated by PCR.

**Results:**

Forty-one colonies of staphylococci were isolated, and six species were identified, including *Staphylococcus sciuri* (61%), *Staphylococcus pasteuri* (15%), *Staphylococcus cohnii* (10%), *Staphylococcus aureus* (7%), *Staphylococcus warneri* (5%), and *Staphylococcus haemolyticus* (2%). Staphylococci were highly resistant to two drug classes, penicillin (93%) and cephalosporin (51%). About 56% of the isolates were methicillin-resistant staphylococci (MRS), and the majority was *S. sciuri* (82%), which is primarily found in horses. Most MRS (82%) were multidrug-resistant. Almost all (96%) of the *mecA*-positive MRS harbored the *blaZ* gene. Almost all MRS isolates possessed an unknown type of SCC*mec*. Interestingly, the AMR rate was notably lower in fighting bulls and cocks than in riding horses, which may be related to the owner’s preference for herbal therapy over antimicrobial drugs.

**Conclusion:**

This study presented many types of staphylococci displayed on bulls, cocks, and horses. However, we found a high prevalence of MRS in horses that could be transmitted to owners through close contact activities and might be a source of AMR genotype transmission to other staphylococci.

## Introduction

Antimicrobial resistance (AMR) is a major public health concern on a global scale [1], and it has a significant economic and health impact because it can result in death and disability [2]. It is considered that misuse and overuse of antimicrobials are the primary factors contributing to AMR development [3,4]. Most AMR studies in animals focus on livestock because it is believed that antimicrobial drugs are extensively used [5-9]. However, studying AMR in pet animals is critical since many common drugs used in humans are also used in pets, which can accelerate the resistance of bacteria to common antimicrobial drugs used in both humans and pets [10-12]. Moreover, the close contact between humans and pets increases the risk of transmission of AMR bacteria between them [13]. Some large animals and livestock in Thailand, such as horses, cattle, and chickens, are occasionally kept as pets for pleasure and sport. Riding horses, fighting bulls, and fighting cocks are regarded as valuable pets in South Thailand, as well as sports animals; these animals have been a part of the Thai community for centuries, associated with culture, and, more recently, used in tourism [14-17]. Sports animals are raised for pleasure and contribute considerably to family income due to high demand in niche markets [14].

Staphylococci are a large genus of commensals isolated from animal skin and mucous membranes. These bacteria have the potential to evolve into opportunistic pathogens able to cause severe diseases in humans and animals [18,19]. Coagulase-positive staphylococci (CoPS) (i.e., *Staphylococcus aureus* and *Staphylococcus pseudintermedius*) are commonly implicated in pathologic processes, whereas coagulase-negative staphylococci (CoNS) (i.e., *Staphylococcus epidermidis*) are considered to be less frequent causes of diseases in humans and animals, both can be zoonotic pathogens [18,19]. Among staphylococci, *S. aureus* is the most concerning species because it impacts on human and animal health. *S. aureus* commonly causes mastitis in cattle [19,20], skin/mucosal infection in chickens [21], and pneumonia in horses [22]. In humans, *S. aureus* is mostly responsible for skin and soft-tissue infections, but it also can infect many internal organs, often with fatal consequences [23]. Furthermore, *S. aureus* can cause food poisoning through its production of enterotoxins and other superantigens [24]. The existence of methicillin-resistant *S. aureus* is a serious public health concern since it is associated with significant morbidity, mortality, length of stay, and financial burden [25]. Furthermore, *S. pseudintermedius* is the second most concerning staphylococci in animals and humans, it is usually found on canine skin or mucosa and frequently causes skin infections [26,27]. This pathogen can be transmitted to humans, resulting in skin and systematic infection [28]. It is not only in dogs but *S. pseudintermediu*s can also be found in cattle and horses [29,30]. For CoNS, *S. epidermidis* is frequently associated with subclinical mastitis in cattle [22].

Due to the fact that sports animals are more prone to injury and illness than other animals, we hypothesize that antimicrobial drugs may be utilized extensively to treat them, resulting in a high incidence of AMR. Therefore, this study aimed to determine the staphylococci diversity and antimicrobial profile in sports animals (riding horses, fighting bulls, and fighting cocks). AMR staphylococci are zoonotic, which means that they can spread from animal to human through close contact activities such as training, handling, and feeding. At present, there are no reports of AMR in sports animals in Thailand, and this research will help raise awareness about AMR in sports animals and humans.

## Materials and Methods

### Ethical approval

This research project was approved by the Institutional Animal Care and Use Committee of Walailak University (ID: 63023).

### Study period and location

The study was conducted from January to May 2021 in Nakhon Si Thammarat province, southern Thailand.

### Animals

The criteria for selecting animals were healthy and mature and had already participated in sports. Therefore, fighting bulls aged 4-6 years, riding horses aged 5-15 years (all sexes), and fighting cocks aged 8-10 months were considered.

For fighting bulls (n=57) and riding horses (n=33), samples were collected with a cotton bud by nasal swabbing. For fighting cocks (n=32), samples were collected by skin swabbing at the skin beneath the wing. After swabbing, samples were dipped in 0.9% sodium chloride in the tube and kept in the foam box before being transferred to tryptic soy broth (TSB) in the laboratory for culture.

### Isolation of staphylococci

Samples in TSB were incubated at 37°C for 24 h. Then, 0.1 mL of the TSB was placed on mannitol salt agar (MSA) and incubated at 37°C for 24 h. All staphylococci-like colonies were cultured again with MSA plus oxacillin (0.5 μg/mL) to primarily identify methicillin-resistant bacteria. Staphylococci-like colonies were selected for purification and investigated for the type of hemolysin on blood agar. Finally, the species of staphylococci were identified using the VITEK^®^ 2 card for Gram-positive organisms in conjunction with the VITEK^®^ 2 COMPACT machine (bioMérieux, Marcy l’Etoile, France).

### Antimicrobial susceptibility test

The antimicrobial susceptibility test was evaluated using the minimum inhibitory concentration (MIC)-based automated system, the VITEK^®^ 2 AST-GN80 test kit cards, and VITEK^®^ 2 COMPACT machine (bioMérieux, Marcy l’Etoile, France) [31,32]. The antimicrobial resistance was determined using an advanced Expert System™ based on the global Clinical and Laboratory Standards Institute guidelines and natural resistance guidelines.

Drug that used for MIC (μg/mL) composed of benzylpenicillin (0.125, 0.25, 1, 2, 8, and 64), oxacillin (0.5, 1, and 2), cefalotin (4, 8, and 32), cefovecin, ceftiofur (0.5, 2, and 4), kanamycin (32, 64, and 128), enrofloxacin (1 and 2), marbofloxacin (0.5 and 2), pradofloxacin (0.125, 0.5, and 2), erythromycin (0.25, 0.5, and 2), clindamycin (CM 0.5 and CM/E 0.25/0.5), doxycycline (0.25, 1, 2, and 4), tetracycline (0.5, 1, and 2), nitrofurantoin (16, 32, and 64), chloramphenicol (2, 8, and 16), and sulfamethoxazole/trimethoprim (152/8, 304/16, and 608/32).

### Detection of AMR genes

Three AMR genes were examined, including *mecA*, *mecC*, and *blaZ* [33-35]. Primers were synthesized by Macrogen (Geumcheon-gu, Seoul, South Korea), and primer sequences are shown in Table-1. All primers were checked for positive DNA before performing the experiments.

**Table-1:** The list of PCR primers.

Gene name	Primer sequence	Product size (bp)	Temperature (°C)
AMR gene			
*mecA*	F: AAAATCGATGGTAAAGGTTGGC	532	56
	R: AGTTCTGCAGTACCGGATTTGC		
*mecC*	F: GAAAAAAAGGCTTAG AACGCCTC	631	56
	R: GAAGATCTT TTCCGTTTTCAGC		
*blaZ*	F: ACTTCAACACCTGCTGCTTTC	173	54
	R: TGACCACTTTTATCAGCAACC		
M-PCR 1 for amplification of *ccr* gene complex with *mecA*			57
*mA1*	GCTATCCACCCTCAAACAGG	286	
*mA2*	ACGTTGTAACCACCCCAAGA		
*α1*	AACCTATATCATCAATCAGTACGT	695	
*α2*	TAAAGGCATCAATGCACAAACACT	937	
*α3*	AGCTCAAAAGCAAGCAATAGAAT	1791	
*Βc*	ATTGCCTTGATAATAGCCITCT		
*α4.2*	GTATCAATGCACCAGAACTT	1287	
*β4.2*	TTGCGACTCTCTTGGCGTTT		
*γR*	CGTCTATTACAAGATGTTAAGGATAAT	518	
*γF*	CCTTTATAGACTGGATTATTCAAAATAT		
M-PCR 2 for amplification of *mec* gene complex class			60
*mI6*	CATAACTTCCCATTCTGCAGATG	1963	
*IS7*	ATGCTTAATGATAGCATCCGAATG	2827	
*IS2*	TGAGGTTATTCAGATATTTCGATGT		
*mA7*	ATATACCAAACCCGACAACTACA	804	

PCR=Polymerase chain reaction

One purified colony was collected for DNA extraction, and bacterial DNA was extracted using the Presto™ Mini gDNA Bacteria Kit (Geneaid, New Taipei City, Taiwan). The PCR mixture was composed of 6.25 mL of KAPA2G Fast HotStart ReadyMix (Roche, Basel, Switzerland), 0.625 mL of each primer (10 pmol/mL), 3 mL of DNase-free water, and 2 mL of DNA template. The PCR programming was as follows: Activation at 92°C for 3 min, denaturation at 92°C for 1 min, annealing at 54-56°C for 1 min, the PCR was performed for 30 cycles before the final extension step at 72°C for and 3 min. The PCR machine was a GeneAmp PCR System 9700 (Thermo Fisher Scientific, MA, USA). The PCR products were confirmed using a commercial sequencing service (Wardmedics, Bangkok, Thailand).

### Staphylococcal cassette chromosomal *mec* (SCC*mec*) typing

The SCC*mec* detection was performed using multiplex PCR (M-PCR) [36], and the primers are shown in Table-1. For M-PCR 1, PCR mixture was 12.5 μL of Master mix, 0.5 μL of each primer, 0.85 μL of MgCl_2_, 2.65 μL of DNase-free water, and 4 μL of DNA. The PCR program was activation step for 2 min at 94°C, 30 cycles of 2 min at 94°C for the denaturation step, 1 min at 57°C for the annealing step, and 2 min at 72°C for the extension step.

For M-PCR 2, PCR mixture for bacterial identification was composed of 12.5 μL of Master mix, 0.5 μL of each primer, 0.5 μL of MgCl_2_, 6.5 μL of DNase-free water, and 3 μL of DNA template. The PCR program was activation step for 2 min at 94°C, 30 cycles of 2 min at 94°C for the denaturation step, 1 min at 60°C for the annealing step, and 2 min at 72°C for the extension step.

The PCR products were verified using an electrophoresis set (Major Science, CA, USA) with a 1.5% agarose gel (Vivantis, Shah Alam, Malaysia) at 135 V/cm for 20 min. In addition, DNA was stained with FluoroVue™ (SmobioHsinchu City, Taiwan) and imaged by the G-BOX F3 Gel imaging machine (G-BOX F3, Syngene, Cambridge, UK). Finally, the PCR products were confirmed using a commercial sequencing service (Wardmedics, Bangkok, Thailand).

## Results

### Isolation of bacteria

Overall, 41 staphylococci colonies were isolated. Twelve colonies were isolated from 32 (37.5%) fighting cocks. Eleven colonies were obtained from 57 (19.3%) fighting bulls, and 18 colonies were purified from 33 (54.5%) horses. Six species of staphylococci were identified from all animals (Table-2). The main population was *Staphylococcus sciuri* (61.0%, n=25), followed by *Staphylococcus pasteuri* (14.6%, n=6), *Staphylococcus cohnii* (9.8%, n=4), *S. aureus* (7.3%, n=3), *Staphylococcus warneri* (4.9%, n=2), and *Staphylococcus haemolyticus* (2.4%, n=1). For each animal, the major staphylococci in cocks were *S. pasteuri*, which accounted for 50% (n=6/12), while *S. sciuri* was the main staphylococci for bulls (81%, n=9/11) and horses (72.2%, n=13/18).

**Table-2:** The number of staphylococcal colonies in which the species was identified.

	*S. sciuri*	*S. warneri*	*S. pasteuri*	*S. haemolyticus*	*S. aureus*	*S. cohnii*	Total
Cocks	3	1	6	1	1	0	12
Bulls	9	1	0	0	1	0	11
Horses	13	0	0	0	1	4	18
Total	25 (61.0%)	2 (4.9%)	6 (14.6%)	1 (2.4%)	3 (7.3%)	4 (9.8%)	41 (100%)

*S. sciuri=Staphylococcus sciuri, S. pasteuri=Staphylococcus pasteuri, S. cohnii=Staphylococcus cohnii, S. aureus=Staphylococcus aureus, S. warneri=Staphylococcus warneri, S. haemolyticus=Staphylococcus haemolyticus*

### AMR profile among sports animals

**Figure-1:**
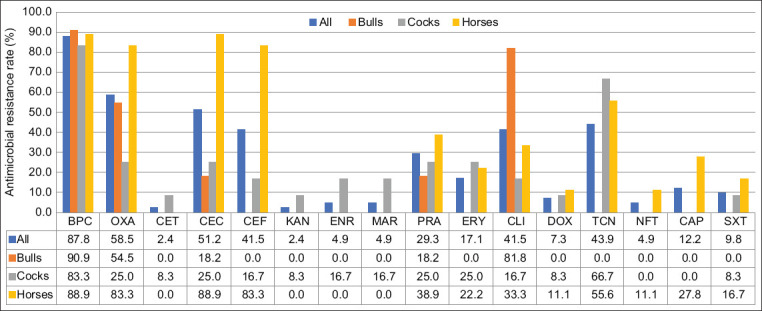
The antimicrobial-resistant profiles of staphylococci in sports animals (n=41). Tested drugs were benzylpenicillin (BPC), oxacillin (OXA), cefalotin (CET), cefovecin (CEC), ceftiofur (CET), kanamycin (KAN), enrofloxacin (ENR), marbofloxacin (MAR), pradofloxacin (PRA), erythromycin (ERY), clindamycin (CLI), doxycycline (DOX), tetracycline (TCN), nitrofurantoin (NFT), chloramphenicol (CAP), and sulfamethoxazole/trimethoprim (SXT).

The AMR profile of all staphylococci is shown in Figure-1. In general, staphylococci were resistant to all drugs tested except kanamycin (n=15/16). Staphylococci obtained from horses, cocks, and bulls were resistant to 12, 11, and 5 drugs, respectively. The antimicrobial drugs with the high rate (>50%) of AMR bacteria were benzylpenicillin (87.8%), oxacillin (58.5%), and cefovecin (51.2%).

Staphylococci obtained from horses exhibited a higher rate of antimicrobial resistance than other animals; they were highly resistant to benzylpenicillin (88.9%), cefovecin (88.9%), oxacillin (88.3%), ceftiofur (83.3%), and tetracycline (55.6%). Staphylococci isolated from bulls were highly resistant to benzylpenicillin (90.9%), CM (81.8%), and oxacillin (54.5%), while those collected from cocks were highly resistant to benzylpenicillin (83.3%) and tetracycline (66.7%).

The resistance to the fluoroquinolone group, enrofloxacin, and marbofloxacin was found only in cocks, while the resistance to doxycycline, nitrofurantoin, and chloramphenicol was found only in horses. The most sensitive drugs (no resistance) for staphylococci isolated from horses were cefalotin, kanamycin, enrofloxacin, and marbofloxacin. The most sensitive drugs for staphylococci isolated from cocks were nitrofurantoin and chloramphenicol. In contrast, cefalotin, ceftiofur, kanamycin, enrofloxacin, marbofloxacin, erythromycin, doxycycline, tetracycline, nitrofurantoin, chloramphenicol, and sulfamethoxazole/trimethoprim were the most sensitive drugs for staphylococci isolated from bulls.

### AMR between *S. sciuri* and non-*S. sciuri*

**Figure-2:**
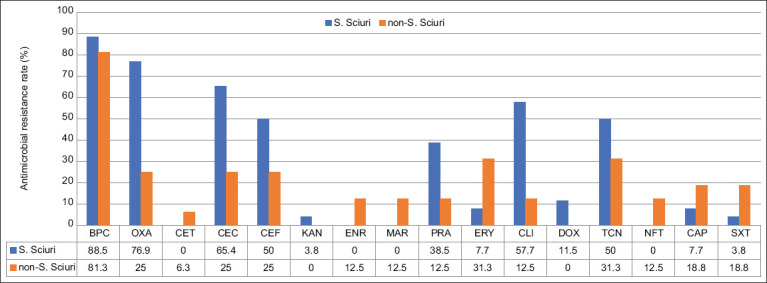
Antimicrobial-resistant profile of *Staphylococcus sciuri* (n=25) compared to non-*S. sciuri* (n=16). Tested drugs were benzylpenicillin (BPC), oxacillin (OXA), cefalotin (CET), cefovecin (CEC), ceftiofur (CET), kanamycin (KAN), enrofloxacin (ENR), marbofloxacin (MAR), pradofloxacin (PRA), erythromycin (ERY), clindamycin (CLI), doxycycline (DOX), tetracycline (TCN), nitrofurantoin (NFT), chloramphenicol (CAP), and sulfamethoxazole/trimethoprim (SXT).

A comparison of AMR rates between *S. sciuri* (n=25) and non-*S. sciuri* (n=16) is shown in Figure-2. The resistance to benzylpenicillin was similar between *S. sciuri* (88.5%) and non-*S. sciuri* (81.3%). Notably, the AMR rate was higher in *S. sciuri* than in non-*S. sciuri* for many drugs, including oxacillin (76.9% vs. 25.0%), cefovecin (65.4% vs. 25.0%), ceftiofur (50.0% vs. 25.0%), CM (57.7% vs. 12.5%), and tetracycline (50.0% vs. 31.3%). Non-*S. sciuri* isolates were more resistant to sulfamethoxazole, trimethoprim, chloramphenicol, nitrofurantoin, erythromycin, marbofloxacin, and enrofloxacin than *S. sciuri* isolates; however, the AMR rate in these drugs was <32%.

### Methicillin-resistant staphylococci (MRS)

MRS was identified by the resistance of oxacillin and the presence of the *mecA* gene, MRS isolates were found at 53.7% (n=22/41), and they were commonly found in horses (68.2%, n=15/22) (Table-3). Methicillin-resistant *S. sciuri* (MRSS) was the most prevalent MRS (81.8%, n=18/22), and the majority of MRSS colonies were recovered from horses (66.7%, n=12/18). In addition, three colonies were methicillin-resistant *S. cohnii* (MRSC) obtained from horses, and one colony was methicillin-resistant *S. haemolyticus* (MRSH) obtained from a cock. Comparison among sports animals, staphylococci collected from horses (83.3%, n=15/18) had a higher rate of MRS than bulls (36.4%, n=4/11) and cocks (25%, n=3/12).

**Table-3:** Antimicrobial genes (*mecA, mecC,* and *blaZ*) and chromosomal *mec* (SCC*mec*) type in methicillin-resistant staphylococci.

No.	Animal	Bacteria	*mecA*	*mecC*	*blaZ*	SCC*mec*
1	Cock	*S. sciuri*	+	-	+	Unidentified
2	Cock	*S. sciuri*	+	-	+	Unidentified
3	Cock	*S. haemolyticus*	+	-	+	Type V
4	Bull	*S. sciuri*	+	-	+	Unidentified
5	Bull	*S. sciuri*	+	-	+	Unidentified
6	Bull	*S. sciuri*	+	-	+	Unidentified
7	Bull	*S. sciuri*	+	-	+	Unidentified
8	Horse	*S. sciuri*	+	-	+	Unidentified
9	Horse	*S. sciuri*	+	-	+	Unidentified
10	Horse	*S. sciuri*	+	-	+	Unidentified
11	Horse	*S. sciuri*	+	-	+	Unidentified
12	Horse	*S. sciuri*	+	-	+	Unidentified
13	Horse	*S. sciuri*	+	-	+	Unidentified
14	Horse	*S. sciuri*	+	-	+	Unidentified
15	Horse	*S. sciuri*	+	-	+	Unidentified
16	Horse	*S. sciuri*	+	-	+	Unidentified
17	Horse	*S. sciuri*	+	-	+	Unidentified
18	Horse	*S. sciuri*	+	-	+	Unidentified
19	Horse	*S. sciuri*	+	-	+	Unidentified
20	Horse	*S. cohnii*	+	-	+	Unidentified
21	Horse	*S. cohnii*	+	-	+	Unidentified
22	Horse	*S. cohnii*	+	-	-	Unidentified

*S. sciuri = Staphylococcus sciuri, S. pasteuri = Staphylococcus pasteuri, S. cohnii = Staphylococcus cohnii, S. aureus = Staphylococcus aureus, S. warneri = Staphylococcus warneri, S. haemolyticus = Staphylococcus haemolyticus*

From 41 colonies, the *mecA* gene was positive in 53.7% (n=22), the *blaZ* gene was positive in 51.2% (n=21), but none were positive for the *mecC* gene. About 95% of *mecA*-MRS isolates tested positive for the *blaZ* gene (n=21/22). One isolate (*S. haemolyticus*) was SCC*mec* type V, whereas the remaining colonies were unidentified (Table-3).

**Figure-3:**
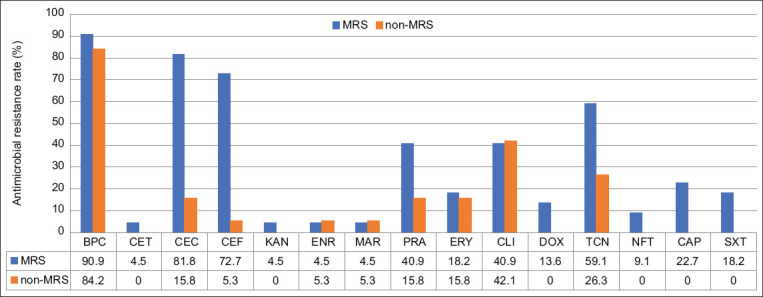
Antimicrobial-resistant profile of MRS (n=22) compared to non-MRS (n=19). Tested drugs were benzylpenicillin (BPC), oxacillin (OXA), cefalotin (CET), cefovecin (CEC), ceftiofur (CET), kanamycin (KAN), enrofloxacin (ENR), marbofloxacin (MAR), pradofloxacin (PRA), erythromycin (ERY), clindamycin (CLI), doxycycline (DOX), tetracycline (TCN), nitrofurantoin (NFT), chloramphenicol (CAP), and sulfamethoxazole/trimethoprim (SXT).

The AMR profile of MRS (n=22) compared to non-MRS (n=19) is shown in Figure-3. Together, MRS isolates were resistant to all tested drugs, but they were highly resistant to benzylpenicillin (90.9%), cefovecin (81.8%), ceftiofur (72.7%), and tetracycline (59.1%). For non-MRS strains, they were highly resistant to only benzylpenicillin (84.2%) but very susceptible to many drugs, including cefalotin, kanamycin, doxycycline, nitrofurantoin, chloramphenicol, and sulfamethoxazole/trimethoprim.

### Multidrug resistance (MDR) and extensively drug-resistance (XDR)

MDR is defined as an isolate that is not susceptible to at least one agent in at least three antimicrobial classes, whereas XDR was defined as non-susceptibility to at least one agent in all but two or fewer antimicrobial categories (i.e., bacterial isolates remain susceptible to only one or two classes) [37,38]. The antimicrobial drugs tested in this study were categorized into nine classes according to the Centers for Disease Control and Prevention [39]. The AMR rate for the penicillin class was 92.7%, the cephalosporin class was 51.2%, the tetracycline class was 43.9%, the lincosamide class was 41.5%, the fluoroquinolone class was 31.7%, the macrolide class was 17.1%, the chloramphenicol class was 12.2%, the trimethoprim/sulfamethoxazole class was 9.8%, the urinary anti-infective class was 4.9%, and the aminoglycoside class was 2.4%.

The number of MDR staphylococci isolates is shown in Table-4. The overall MDR rate was 56.09% (n=23/41); the highest rate of MDR was observed in *S. sciuri* (72.00%). The majority of MDR isolates were obtained from horses (88.89%). MDR isolates were mostly MRS strains (81.82%). The XDR was found in one colony of *S. sciuri* isolated from cock, and it was resistant to eight of nine drug classes.

**Table-4:** Number of MDR staphylococci colonies.

	Animals			Bacteria		MRS	
		
	Bull	Horse	Cock	*S. sciuri*	Non-*S. sciuri*	MRS	Non-MRS
MDR colonies	3	16	4	18	5	18	5
Total colonies	11	18	12	25	16	22	19
% MDR	27.27%	88.89%	33.33%	72.00%	31.25%	81.82%	26.32%

MDR=Multidrug resistance, *S. sciuri=Staphylococcus sciuri*

## Discussion

The present study is the first to identify the diversity and antimicrobial resistance profile of staphylococci in sports animals in Thailand; in particular, no country has reported AMR in fighting bulls or fighting cocks. We revealed that *S. sciuri* is the most common staphylococci found in sports animals, particularly horses. Most isolates were -resistant and MDR, particularly penicillin, cephalosporin, and tetracycline. Almost all MRS also carried the *mecA* and *blaZ* genes with an unknown type of *SCCmec*.

The coagulase-negative species *S. sciuri* is an opportunistic pathogen, typically found in many habitats, including animals, humans, and the environment [40-43]. *S. sciuri* is an important human pathogen responsible for endocarditis, peritonitis, urinary tract infections, wound/skin infections, and septic shock [43-47]. Infections of *S. sciuri* in human hospitals are emerging and increasing in several countries [48-51], which may result from the high adaptation capacity to the environment [52]. In animals, *S. sciuri* can cause fatal exudative epidermitis in piglets [53], wound infection in horses [54], and mastitis in cattle [55]. At present, the AMR profiles of *S. sciuri* in animals are rarely reported; studies in Belgium and Denmark have demonstrated that most *S. sciuri* isolates are not MRRS, but they are susceptible to cephalosporin [56,57]. Conversely, the prevalence of MRSS was very high in this present study (>70%), and they were highly resistant to cephalosporin and other drug classes. Notably, the high proportion of MRRS strains was derived from horses, and these findings may be explained by the fact that cephalosporin is a widely used drug in Thailand for treating skin and musculoskeletal diseases in horses (personal communication) and is also the second most consumed antimicrobial agent globally [58].

*S. cohnii* was the second most often observed species in this study, and some strains were MRS and MDR; however, knowledge about this species is very limited. The previous studies show that *S. cohnii* can be found in humans and other animals such as dogs, cattle, horses, and chickens [59-62]. *S. cohnii* isolated from dairy cows harbors various AMR genes, and it is resistant to a wide range of antimicrobial drugs [63]. The infection of *S. cohnii* in humans is rare and is related to opportunistic infection [61,64]. MDR *S. cohnii* isolates are recently detected (5%) on frequently touched surfaces in a London hospital [49]. In the same way as *S. sciuri*, *S. cohnii* may serve as an AMR gene reservoir for harmful staphylococcal species.

*S. sciuri* has gained attention as a natural reservoir for the *mecA* gene, which is believed to be a precursor to the methicillin resistance gene seen in other staphylococci [42,49,65]. Staphylococci can possess the *mecA, mecC* and *blaZ* genes, which are all significantly associated with the methicillin-resistant phenotype due to these genes control enzymes involved in the removal and destruction of beta-lactam antimicrobial drugs [66,67]. In the present study, more than 70% of *S. sciuri* carried *mecA* but not *mecC*, which is higher than a previous study (46%) [51]. However, *S. sciuri* carries the *mecC* gene has been reported in ruminants, horses, and chickens [68-70]. Moreover, the coexistence rate of the *mecA* and *blaZ* genes in *S. Sciuri* was extremely high (> 90%) in this study, compared to a previous study [71], raising serious concern for AMR in this species because the *mecA* and *blaZ* genes coexist often in other staphylococci species, including *S. pseudintermedius* and *S. aureus* [72-76]. The majority of MRSS in this study also had an MDR phenotype which is rarely reported; MDR staphylococci are mainly *S. aureus* [77-80]. MRSS carrying multiple AMR genes might threaten animal and human health because this pathogen is capable of causing a variety of diseases and transmit AMR genes to other staphylococci [43-47,53-55]. Interspecies horizontal gene transfer from *S. sciuri* to others could occur through many mechanisms, but bacteriophage has been shown to be one such mechanism [81].

In this study, staphylococci were resistant to penicillin at a higher proportion (90%) than other drugs. This finding is consistent with other studies conducted in Thailand that discovered a significant prevalence of penicillin-resistant CoNS and *S. aureus* in dairy cows and pigs [82,83]. This finding is unsurprising, given we discovered that penicillin is the most frequently used antimicrobial drug in sports animals in Thailand (personal communications) and the most commonly used antimicrobial agent in veterinary medicine both in Thailand and throughout the world [58,84].

Another drug, tetracycline, is the most sold antimicrobial drug globally [85] and is one of the top five antimicrobial drugs used in food animals in Thailand [84]. In the present study, the overall incidence of tetracycline resistance was not high; however, the AMR rate was increased in *S. sciuri* (50%) and bacteria isolated from fighting cocks (67%). The tetracycline-resistant rate of *S. sciuri* in this study is similar to a previous study in Belgium [57] but higher than in Denmark [56]. The high AMR rate of isolates obtained from fighting cocks may be associated with the fact that the tetracycline drug class is routinely used in fighting cocks (personal communication) and broilers [86] to treat and prevent bacterial diseases. As a result, *S. aureus* obtained from meat (chicken and pork) in Thailand mostly exhibit phenotypes that are resistant to tetracycline (80-90% resistant rate) [87,88].

In this study, the AMR rate of fighting bulls and cocks was generally lower than that of horses. One possible explanation for this is that the owners of fighting bulls and fighting cocks usually rely on herbal medicine rather than modern medicine (personal communication). For example, turmeric (*Curcuma longa*) extract is commonly used to treat wounds in fighting bulls and fighting cocks (personal communication); this extract has antimicrobial activity and improves wound healing [89,90].

## Conclusion

This study presented many types of staphylococci displayed on fighting bulls, fighting cocks, and horses. We found a high proportion of MRS in horses, but not in fighting bulls and fighting cocks, presumably because owners prefer to use herbs instead of antimicrobial drugs. These MRS are MDR bacteria and carry multiple AMR genes that can potentially cause infection in humans and animals and might be a source of transmission of the drug-resistant genotype to other staphylococci. The drug-resistant data from this study will help clinicians with animal treatment in the future and raise a concern about using antimicrobials in sports animal practice, particularly in horses. This study area is limited only to Nakhon Si Thammarat province, so further studies in different areas should be investigated to identify the prevalence of AMR in the country.

## Authors’ Contributions

PF, RN, NS, CS, YP, TT, and TW: Collected samples. PF, RN, NS, TT, and KB: Cultured bacteria, molecular analysis, and microbial sensitivity. CS, YP, and TW: Communicated and interviewed with farmers. TW and PF: Designed the experiment. TW: Received the grant and managed the project. TW and PF: Drafted the manuscript, and all authors edited and approved the final manuscript. All authors read and approved the final manuscript.
